# Putative mobilized colistin resistance genes in the human gut microbiome

**DOI:** 10.1186/s12866-021-02281-4

**Published:** 2021-07-22

**Authors:** Bruno G. N. Andrade, Tobias Goris, Haithem Afli, Felipe H. Coutinho, Alberto M. R. Dávila, Rafael R. C. Cuadrat

**Affiliations:** 1 Department of Computer Science, Munster Technological University, MTU/ADAPT, Cork, Ireland; 2 Department of Molecular Toxicology, Research Group Intestinal Microbiology, German Institute of Human Nutrition Potsdam-Rehbruecke – DIfE, Arthur-Scheunert-Allee 114-116, 14558 Nuthetal, Germany; 3 Departamento de producción vegetal y microbiología, Universidad Miguel Hernández, Alicante, Spain; 4 Computational and Systems Biology Laboratory and Graduate Program on Biodiversity and Health, Oswaldo Cruz Institute, FIOCRUZ, Rio de Janeiro, RJ Brazil; 5 Bioinformatics and Omics Data Science, Berlin Institute for Medical Systems Biology (BIMSB), Max Delbrück Center (MDC), Berlin, Germany; 6 Department of Molecular Epidemiology, German Institute of Human Nutrition Potsdam-Rehbruecke, Arthur-Scheunert-Allee 114-116, 14558 Nuthetal, Germany

**Keywords:** Colistin, Human microbiome, MCR, Metagenomics, Antibiotic resistance genes

## Abstract

**Background:**

The high incidence of bacterial genes that confer resistance to last-resort antibiotics, such as colistin, caused by mobilized colistin resistance (*mcr*) genes, poses an unprecedented threat to human health. Understanding the spread, evolution, and distribution of such genes among human populations will help in the development of strategies to diminish their occurrence. To tackle this problem, we investigated the distribution and prevalence of potential mcr genes in the human gut microbiome using a set of bioinformatics tools to screen the Unified Human Gastrointestinal Genome (UHGG) collection for the presence, synteny and phylogeny of putative mcr genes, and co-located antibiotic resistance genes.

**Results:**

A total of 2079 antibiotic resistance genes (ARGs) were classified as *mcr* genes in 2046 metagenome assembled genomes (MAGs), distributed across 1596 individuals from 41 countries, of which 215 were identified in plasmidial contigs. The genera that presented the largest number of *mcr*-like genes were *Suterella* and *Parasuterella*. Other potential pathogens carrying *mcr* genes belonged to the genus *Vibrio*, *Escherichia* and *Campylobacter*. Finally, we identified a total of 22,746 ARGs belonging to 21 different classes in the same 2046 MAGs, suggesting multi-resistance potential in the corresponding bacterial strains, increasing the concern of ARGs impact in the clinical settings.

**Conclusion:**

This study uncovers the diversity of *mcr*-like genes in the human gut microbiome. We demonstrated the cosmopolitan distribution of these genes in individuals worldwide and the co-presence of other antibiotic resistance genes, including Extended-spectrum Beta-Lactamases (ESBL). Also, we described *mcr*-like genes fused to a PAP2-like domain in *S. wadsworthensis*. These novel sequences increase our knowledge about the diversity and evolution of *mcr*-like genes. Future research should focus on activity, genetic mobility and a potential colistin resistance in the corresponding strains to experimentally validate those findings.

**Supplementary Information:**

The online version contains supplementary material available at 10.1186/s12866-021-02281-4.

## Background

The prevalence of antibiotic resistance (AR) in clinical pathogens is a significant public health concern, especially in low and middle-income countries (LMICs) [1]. The misuse of antibiotics is the main driving factor for the rise of antibiotic-resistant bacteria. Still, its importance is often underestimated in community infections, as hospitalized infections gain the most attention [2]. Many previous studies have addressed the prevalence of AR in clinical environments [3–5], and more recently AR prevalence in non-hospitalized populations too [6, 7]. Most of these studies were conducted on cultivable clinical strains using microbiological methods that involve cultivation and antibiogram tests. However, the advances in high-throughput sequencing and bioinformatics provided access to the human resistome through the analysis of metagenomes and metagenome-assembled genomes.

The resistome is defined as the collection of the antibiotic resistance genes (ARGs) in a single microorganism, or in a microbial community, and has been investigated in different environments, such as soils [8], oceans [9], and host-associated microbiomes such as the animal [10] or human [11] gut. Understanding the human resistome in hospitalized and non-hospitalized populations is essential because the commensal microbiota can host ARGs and transfer it from and to pathogenic bacteria through horizontal gene transfer (HGT) [12], e.g., during an infection. In addition, HGT can also play a role in ARG mobilization to environmental communities by water and soil contamination [13] or the food we ingest [14, 15]. The gut microbiome is of particular interest in the investigation of ARGs in the human microbiota since it is the largest, most diverse [16], highly exposed and affected by the intake of antibiotics. A potential influx of ARGs can occur via food intake and/or unhygienic conditions, and the efflux of ARGs to wastewater plants enhances the spread to other environments. As such, the human gut microbiome is thought to be responsible for transferring ARGs [17] to the environment to a large extent. Therefore, the search for ARGs in the human gut microbiome, mostly performed using metagenomics and culturomics approaches, is one of the key fields to unravel the transfer of ARGs and the evolution of antibiotic resistance in bacteria. While past metagenomic studies on ARGs relied on shorter contigs, often below 50 kilobases, new assembly methods that allow the recovery of nearly complete bacterial genomes have been developed. Such methods have been applied to study the human gut microbiome and led to the recovery of thousands of metagenome-assembled genomes (MAGs) [18–20]. These datasets were recently combined into one resource, the Human Gastrointestinal Bacteria Genome Collection (HGG) [21], a valuable resource for ARG screenings. The advantage of MAGs versus traditional metagenome gene catalogs is manifold; the most apparent is the high accuracy of phylogenetic affiliations and often complete gene clusters, revealing gene synteny. The latter is of high interest when studying ARGs since the genetic environment often reveals the potential for the genetic mobility of ARGs, e.g., their location on genetic islands or plasmids [22]. Besides, it is also possible to investigate the presence of multi-drug-resistant (MDR) bacteria by detecting more than one ARG in the same bacterial genome or contig [9] when using the MAG based approach.

Recently, colistin (Polymyxin E) has gained attention as the last line of defense drug against MDR bacteria, especially carbapenem-resistant gram-negative pathogens [23]. However, reports of colistin-resistant bacteria are becoming more frequent [24], with its prevalence reaching as high as approximately 20–40% among Carbapenem-Resistant *Klebsiella pneumoniae* (CRKP) in Italy and Greece [25]. In the past, the only known acquired resistance mechanism for colistin was mediated by chromosomal mutations, mainly in genes regulating the chemical additions of L-Ara4N and pEtN [26]. The first plasmid-mediated polymyxin resistance gene, designated mobilized-colistin resistance-1 (*mcr*-1), was described in *Enterobacteriaceae* in 2006 [27]. Later it was followed by the additional mcrs, *mcr*-2 [28], *mcr*-3 [29], *mcr*-4 [30], *mcr*-5 [31] and, recently, *mcr*-6 to *mcr*-10 [32–36]. An intrinsic *mcr*-1-like homolog from *Moraxella osloensis* was described, and named icr-Mo [37], raising the discussion about possible origins of *mcr* in *Moraxella*. The spread of *mcr* is of public health concern as it has been attributed to colistin’s over-use, especially in livestock [38] and aquaculture [39, 40]. Here, we screened the HGG for the presence of *mcr*-like genes and other ARGS among human gut bacterial genomes.

## Methods

### Data retrieval

We retrieved approximately 171 million non-redundant protein sequences (clustered at 100% in the file uhgp-100.faa) as well as the 276,349 metagenomic assembled genomes (MAGs) and 10,648 genomes from bacterial isolates and the metadata including taxonomic affiliation from the genomes from the Unified Human Gastrointestinal Protein (UHGP) catalog v1.0 [21], part of the HGG, at http://ftp.ebi.ac.uk/pub/databases/metagenomics/mgnify_genomes/human-gut/v1.0/.

### Antibiotic resistance gene screening

Antibiotic-resistance genes were identified using previously described methods applied to a dataset of marine samples [9]. Briefly, we used the deepARG tool [41] (model version 2), a deep learning approach developed to identify ARGs in open reading frames, to search for ARGs in the non-redundant proteins provided by the UHGP catalog, using the Long sequences (LS) model with default parameters. The deepARG model was originally built considering a dissimilarity matrix of all ARGs from 3 different databases, such as the Antibiotic Resistance Genes Database [ARDB], Comprehensive AntibioticResistance Database [CARD], and UniProt, being an alternative to the “best hits” approach. Only genes with estimated probability of ≥0.8 were considered for this study. We then selected all proteins classified as mcr in the deepARG results and explored the prevalence of these putative ARGs in different countries and across diverse taxa.

### Plasmid classification

To verify if the putative genes are of chromosomal or plasmid origin, we analyzed contigs in which sequences classified as MCR were identified using the PlasFlow software [42] with the default threshold of ≥0.7. This software uses neural network models trained on full genomes and plasmid sequences to predict the sequence origin with 96% accuracy.

### Phylogeny

Protein sequences classified as MCR present in contigs identified as plasmids in MAGs and complete genomes, were clustered at 97% of sequence identity with the software cd-hit v4.7 [43] to reduce the redundancy of protein sequences in the tree. The representative sequences of each cluster (and reference sequences obtained at NCBI) were then submitted to NGPhylogeny.fr [44] where the protein sequences were aligned by Mafft [45], then the informative phylogenetic regions were selected by BMGE [46], and the Maximum likelihood (ML) reconstruction was calculated by PhyML 3.0 [47] with the model selection performed by SMS (AIC method) [48], and 100 bootstrap replicates to infer significance.

An additional phylogenetic tree was constructed using all MCR protein sequences (from plasmids, chromosomes and reference databases), clustered at 99% identity with cd-hit v4.7. Sequences were queried against the Pfam database using hmmscan from HMMER 3.3 [49] to identify protein domains. Only sequences that displayed both the complete EptA_B_N and sulfatase domains were kept for subsequent analysis. Next, the sequences of each individual domain were extracted from the complete sequences. Domain sub-sequences were aligned individually through MUSCLE v3.8.1551 [50] with default parameters. Aligned sequences were concatenated and used for phylogenetic reconstruction through IQ-Tree v1.6.12 [51] using the LG + R8 model. Bootstrap analysis was performed with 1000 replicates. The final tree was visualized and decorated using the Interactive Tree of Life (iTOL) software (https://itol.embl.de/).

### Data visualization

For data visualization, we used the python3 libraries pandas and matplotlib, and embedded the methods in a jupyter notebook file for the sake of clarity and reproducibility (See Data and Code availability section).

## Results and discussion

In recent years, culture-independent approaches have been used to investigate the extent, diversity and impact of the resistome from different sources, their natural reservoirs, hosts and genomic context. Herein, we investigate the diversity, abundance and prevalence of mcr genes in human populations stool microbiomes, an important ARG that confers resistance to last-resource antibiotics.

### MCR diversity and distribution in the human gut microbiome

**Fig. 1:**
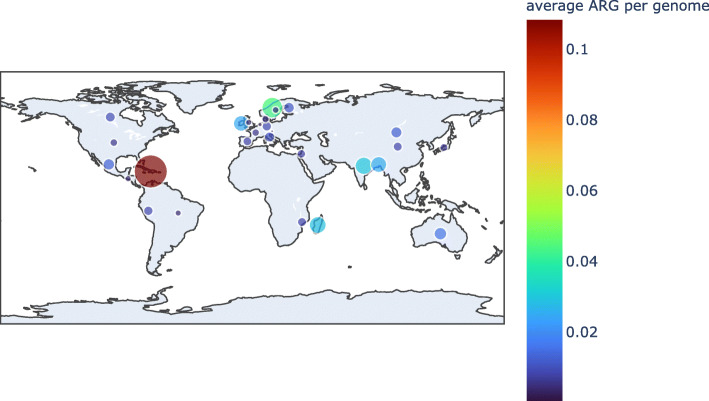
Relative prevalence of mcr-like genes in countries with at least 50 genomes in the study. Bubble area shows the number of genes divided by the number of genomes from each country

We identified a total of 2079 protein sequences classified as MCR (13 MCR-1, 1 MCR-1.2, 9 MCR-2, 634 MCR-3, 456 MCR-4, 966 MCR-5) in 2046 genomes (166 from isolates and 1880 from MAGs), present in 1596 individuals from 41 different countries (7.2% from the total 21,866 in the study). It is important to note the restricted classification of MCR in the deepARG model 2, as it only assigns sequences up to MCR-5. Of the 2079 *mcr*-like genes, 215 (10.34%), were classified by PlasFlow to be located on plasmids, while 1239 MCRs are classified as chromosomally encoded, and 625 genes were in contigs without classification. To illustrate the prevalence and abundance of MCR-like sequences in different countries, we plotted the relative number of genes normalized by the total number of biosamples per country, with Haiti, South Korea, Norway and India identified as having the highest relative number of MCR-like sequences (Fig. 1). However, a quantitative comparison is not possible due to the unbalanced number of genomes from each country (from < 50 to > 50,000), randomness, and collection bias.

### A possible link between *Sutterella* and *Parasutterella* genus, and MCR genes

**Fig. 2:**
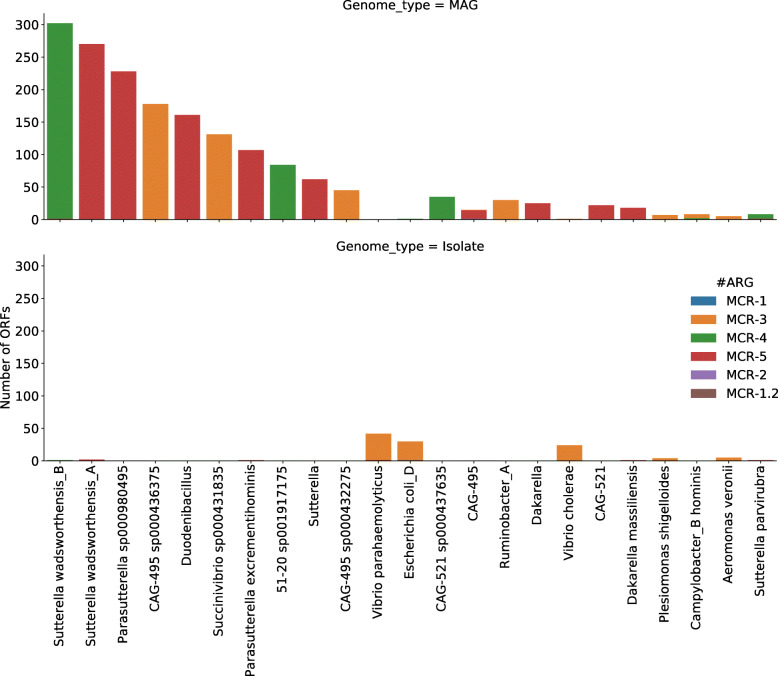
Prevalence of mcr-like genes in different species (with 10 or more mcr-like genes) in MAGs and isolates

The genus *Sutterella* presented the largest number of *mcr*-like genes (667 homologs among 2555 Sutterella genomes in the UHGG), followed by *Parasutterella* (338 genes/1420 genomes) and the alphaproteobacterial genus CAG-495 (258 genes/1181 genomes) (Fig. 2). The genus *Sutterella*, mainly represented by the species *S. wadsworthensis*, is highly prevalent and abundant in the healthy human gut microbiota [52], while not generally considered pathogenic [53, 54]. Nevertheless, a link between this genus and autism in children was suggested [55], and a higher prevalence in prediabetics observed too [56], while the role of *Sutterella* therein remains unknown. Due to isolations from several inflamed body parts, *S. wadsworthensis* might be considered an opportunistic pathogen [57]. While AR has been detected in *S. wadsworthensis*, so far, there has been no report of colistin resistance in the *Sutterella* genus. One study reported a *S. wadsworthensis* strain as susceptible to colistin [58], however, this test was performed by disc diffusion which is known to be error-prone [59]. This suggests that colistin susceptibility of *Sutterella* strains from the human gut must be experimentally validated in the future. Similar to *Sutterella*, the *Parasutterella* genus, while sometimes linked to diseases, appear to be ordinary members of the human gut microbiota [60], pathogenic only in rare cases. The uncharacterized CAG-495 genus was reported to be abundant in patients diagnosed with the Vogt-Koyanagi-Harada disease [61]. Since so far, no reports on *mcr*-like genes in the here-mentioned three genera are published (most probably since there are few studies on human gut MAGs with deepARG), they should be investigated more thoroughly in the future when it comes to the spread of mcr-like genes in the human gut.

### *Mcr*-like genes identified in potential pathogens

The main potentially pathogenic species carrying *mcr*-like genes were isolate strains of *Vibrio parahaemolyticus and V. cholerae*, *Escherichia coli* (only few pathogenic strains, mainly a commensal gut bacterium), and *Campylobacter hominis*, associated with Crohn’s disease and inflammatory bowel disease [62]. Most of these bacterial strains were isolated from sick individuals. MAGs from *Vibrio* species or *E. coli* strains most probably residing as commensal, non-pathogenic in the healthy human gut seem to very rarely contain *mcr*-like genes. Only one of the non-isolate MAGs encoding putative mcrs belonged to *V. cholerae* (GUT_GENOME140393, sample from Bangladesh) and *E. coli* (GUT_GENOME127555, sample from Mongolia). The prevalence of putative mcr genes among *V. cholerae* human gut MAGs is low, with only one occurrence out of 25. The predicted *E. coli* GUT_GENOME127555 *mcr*-gene was classified as *mcr*-4, which contrasts the usually detected *mcr*-1, *mcr*-2 and *mcr*-3 among the *E. coli* isolate genomes and has high similarity (96% identity) to the chromosomally encoded *EptA* of strain K12. A premature stop codon approximately 100 amino acids before the conserved stop codon might preclude the protein from synthesis though. This gene was classified as located in a plasmid by PlasFlow, yet this classification was difficult to verify manually due to the short contig size (4.1 kbp). Hence, it is difficult to predict whether this is a mobilized/plasmid-encoded or chromosomally stable mcr-like gene.

Among the *Campylobacter* genomes, those belonging to *C. hominis* species were all detected among MAGs and, while *C. hominis* is only rarely associated with diseases, the high occurrence of putative *mcr*-3/*mcr*-4 (10 out of 21 overall *C. hominis* MAGs) warrants further investigation. Of the 23 *C. hominis* MAGs in the UHGG catalog, the 10 encoding MCR-like proteins were derived from only two studies pointing to the clustered occurrence of *C. hominis*. Two *mcr*-like genes predicted to be located on plasmids with a high sequence identity to *mcr*-3 like genes were detected in enterobacterial species, but a sequence comparison to recently described *mcr*-10 sequences revealed that they can be assigned to this class, as described for other *Enterobacter* species [32, 63]. Both species from the UHGG, *E. himalayensis* and *E. sesami* (as designated in the genome-taxonomy database used by the UHGG), are described in the literature as *Enterobacter hormaechei* subsp. *hoffmannii* and *E. asburiae*, respectively [64]. Both species are linked to various infections, but their complicated phylogeny and strain-specific pathogenicity makes a specific disease-related prediction difficult. Some strains were reported to be colistin-resistant [65].

### PAP2-MCR fusion proteins

**Fig. 3:**
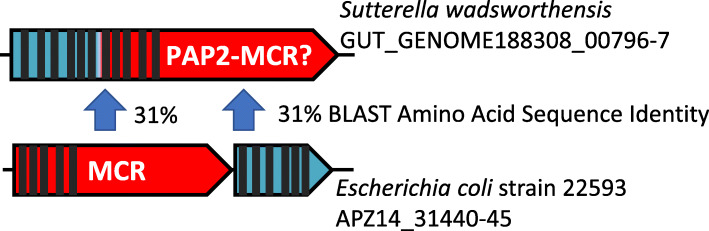
PAP2-domain in mcr-like gene from *Sutterella wadsworthensis*. Comparison with *E. coli mcr*-1 gene including the PAP2-like phosphatase gene downstream. PAP2-domains are colored turquoise, *mcr*-like gene/regions red. Predicted transmembrane helices (TMHMM 2.0 server) are indicated as black bars

A total of 1142, or 54.93% of the MCR-like proteins included a PAP2_like domain at the N-terminal end of their sequence (Fig. 3), indicating an unusual and not characterized fusion between these ORFs. Of the 1142 PAP2-MCR fusion proteins 795 were classified as mcr-5, 333 as mcr-4 and 14 as mcr-3 and most were detected in the genera *Sutterella*, *Parasutterella*, *Duodenibacillus* and the uncultivated CAG-521 genus. Eleven mcr-like genes which were predicted to be located on a plasmid (PlasFlow score ≥ 0.7) contained this PAP2-domain, all of them predicted in *Sutterella wadsworthensis* genomes as homologs to *mcr*-4 genes. While the PAP2-containing domain shares only weak similarity (31% amino acid sequence identity across 50% coverage of the *E. coli* sequence) to the hpap gene, which is located downstream *mcr*-1 of *E. coli*, the number and position of transmembrane helices is conserved (Fig. 3).

Usually, PAP2 is found in a separate ORF downstream of the mcr gene, immediately adjacent to *mcr*-1 and *mcr*-6 genes [66]. However, PAP2 was always described as being encoded by a separate ORF. The encoded phosphatase is suggested to enhance colistin resistance and [67], being crucial for the MCR enzymatic action. The observation of a fused pap2-domain to an *mcr*-like gene was unexpected as this was not reported in any of the literature with regards to this gene. Further physiological studies should shed light on the functionality of these genes as conferring colistin resistance. In addition, this unusual genetic architecture could help classify *mcr*-like genes and might even allow insights into the evolution of *mcr*-like genes, especially in the human gut.

### Multi-resistance potential of genomes carrying *mcr*-like genes

**Fig. 4:**
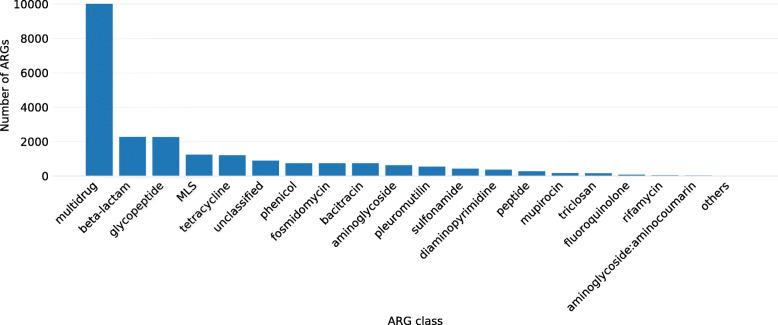
Number of ARGs per class co-occurring in the same genome with Mcr-like genes

To assess and measure the multi-resistance potential of genomes and MAGs carrying *mcr*-like genes, we verified the existence of other ARGs detected by deepARG in genomes containing *mcr*-like genes. We identified a total of 22,746 ARGs (from 21 ARG classes and some unclassified) co-occurring with *mcr*-like genes, among which the most abundant classes were multidrug resistance (10,008 ARGs), beta-lactam (2271 ARGs) and glycopeptide (2261 ARGs) (Fig. 4). However, several of those sequences on the multidrug class are efflux proteins, and as discussed in our previous study [9], those are very hard to distinguish from other transporters that are not involved in antibiotic resistance.

Regarding the beta-lactam group, we identified 1138 penA, a penicillin-binding protein 2 (PBP 2) associated with reduced susceptibility to oral cephalosporins [65], and 673 pbp1-A and 129 pbp1-B that are also penicillin-binding proteins.

In addition, we identified 25 *bla*OXA (class D beta-lactamase capable of hydrolasing 3rd generation cephalosporins) [68], 3 *bla*CTX-M (a plasmid-encoded ESBL found in Enterobacteriaceae, likely acquired from the environmental bacteria *Kluyvera spp.* by HGT [69]), 2 *bla*TEM-153, 7 *bla*TLA-1 and 2 *bla*CFXA-6. The *bla*CTX-M enzymes have been found associated with insertion elements (ISEcp1) and transposable elements (for example, Tn402-like transposons). Many conjugative plasmids can transport these mobile elements, and consequently, these enzymes became the most prevalent ESBL [70, 71]. In *Sutterella wadsworthensis*, the species showing the highest level of mcr-like genes, ARGs related to fosmidomycin (PENA), beta-lactam (rosB) and glycopeptides (vanR) are the most prevalent (see dataset mcr_with_otherARGs_dataframe.tsv). Colistin is a last-resource antibiotic used against MDR bacteria with extended-spectrum beta-lactamases (ESBL), which makes the investigation of the presence of those ARGs in genomes containing MCR-like sequences so important.

### Phylogenetic reconstruction reveals a close relationship between MCR-like and clinical MCR genes

**Fig. 5:**
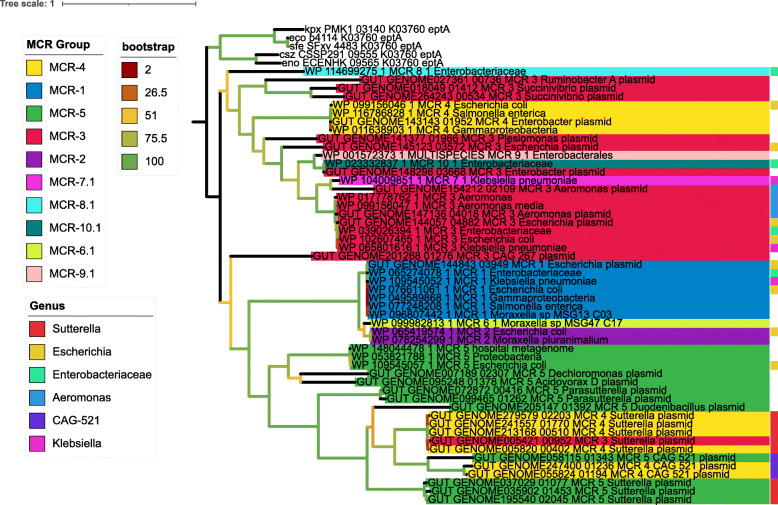
Phylogenetic tree of MCR-like sequences. We used only sequences present in contigs classified as plasmids and we clusterized similar sequences with CD-HIT on 97% similarity. The phylogenetic informative regions were selected by BMGE and the Maximum likelihood (ML) phylogenetic tree was calculated by PhyML 3.0 with the model selection performed by SMS (AIC method) and 100 bootstrap replicates to infer significance. We added clinical MCR sequences from NCBI to the analysis (preceded by an accession number starting with WP). UHGG sequences predicted to be located on a plasmid by PlasFlow contain the “plasmid” note

Phylogenetic reconstruction (Fig. 5) revealed that the protein sequences classified as MCR-4 and MCR-5 from *Sutterella*, *Parasutterella*, and CAG-521 grouped in a single clade with support value of 1, and clinical MCR-5 sequences in an adjacent clade, however these sequences could represent a new group of *mcr*. All the mcr-like sequences used to generate the tree were grouped with clinical MCRs instead of the outgroup eptA, providing additional evidence that those sequences are closer to mcr sequences than to eptA.

An additional phylogenetic reconstruction using all MCR proteins clustered at 99% identity revealed additional patterns regarding the diversity of these genes within the human resistome (Figure S1). Namely, this analysis confirmed the high prevalence of *mcr*-4 among *Sutterella* and *mcr*-5 among *Duodenibacillus*, while *mcr*-3 was widespread among multiple genera. Notably, the classification into MCR groups was not in complete agreement with the topology of the tree. This suggests that there are issues with the approaches used to classify sequences into the recognized groups, which warrants additional care from researchers when classifying these sequences and depositing them on reference databases. Another possibility is that of convergent evolution, which would suggest that MCRs might evolve to resemble the primary structure of MCRs from distinct groups under the correct evolutionary pressure. Another interesting feature revealed by this tree is the lack of separation between chromosome and plasmid encoded MCRs. This finding indicates recombination between chromosomes and plasmids involving MCRs, which seems to be a widespread trait not specific to any particular taxa.

## Conclusions

This study uncovers the diversity of *mcr*-like genes in the human gut microbiome. We demonstrated the cosmopolitan distribution of these genes in human gut samples around the globe and the co-presence of other antibiotic resistance genes, including ESBLs. Also, we described *mcr*-like genes encoded in the same ORF with PAP2-like. Although these novel sequences increase our knowledge about the diversity and evolution of *mcr*-like genes, their activity has to be experimentally validated in the future.

## Supplementary Information

**Additional file 1: Figure S1.** Phylogenetic tree of MCR-like sequences. We clusterized similar sequences with CD-HIT on 99% similarity. Only sequences displaying both the complete EptA_B_N and sulfatase domains were used. Aligned sequences were concatenated and used for phylogenetic reconstruction through IQ-Tree v1.6.12 using the LG + R8 model. Bootstrap analysis was performed with 1000 replicates. The final tree was visualized and decorated using the Interactive Tree of Life (iTOL) software (https://itol.embl.de/).

## Data Availability

All the code used in this study is available at https://github.com/rcuadrat/human_microbiome_mcr and all the data is available at Zenodo (10.5281/zenodo.4399676).

## Abbreviations

AIC: Akaike information criterion
AR: Antibiotics resistance
ARG: Antibiotics resistance gene
BMGE: Block Mapping and Gathering with Entropy
ESBL: Extended-spectrum beta-lactamases
HGT: Horizontal Gene Transfer
HGG: Human Gastrointestinal Bacteria Genome Collection
LMIC: Low and middle-income countries
MCR: Mobilized colistin resistance
MDR: Multi-drug-resistant
MAG: Metagenomic Assembled Genome
ML: Maximum likelihood
NCBI: National Center for Biotechnology Information
ORF: Open reading frame
SMS: Smart Model Selection
UHGG: Unified Human Gastrointestinal Genome
UHGP: Unified Human Gastrointestinal Proteome
