# Supplementary material for: Putative mobilized colistin resistance genes in the human gut microbiome
Source: BMC Microbiol. 2021 Jul 22;21:220. doi: 10.1186/s12866-021-02281-4 (PMC8296556; doi:10.1186/s12866-021-02281-4)
Supplement: Supplementary file 1 — Additional file 1: Figure S1. Phylogenetic tree of MCR-like sequences. We clusterized similar sequences with CD-HIT on 99% similarity. Only sequences displaying both the complete EptA_B_N and sulfatase domains were used. Aligned sequences were concatenated and used for phylogenetic reconstruction through IQ-Tree v1.6.12 using the LG + R8 model. Bootstrap analysis was performed with 1000 replicates. The final tree was visualized and decorated using the Interactive Tree of Life (iTOL) software (https://itol.embl.de/). [file 12866_2021_2281_MOESM1_ESM.pdf]

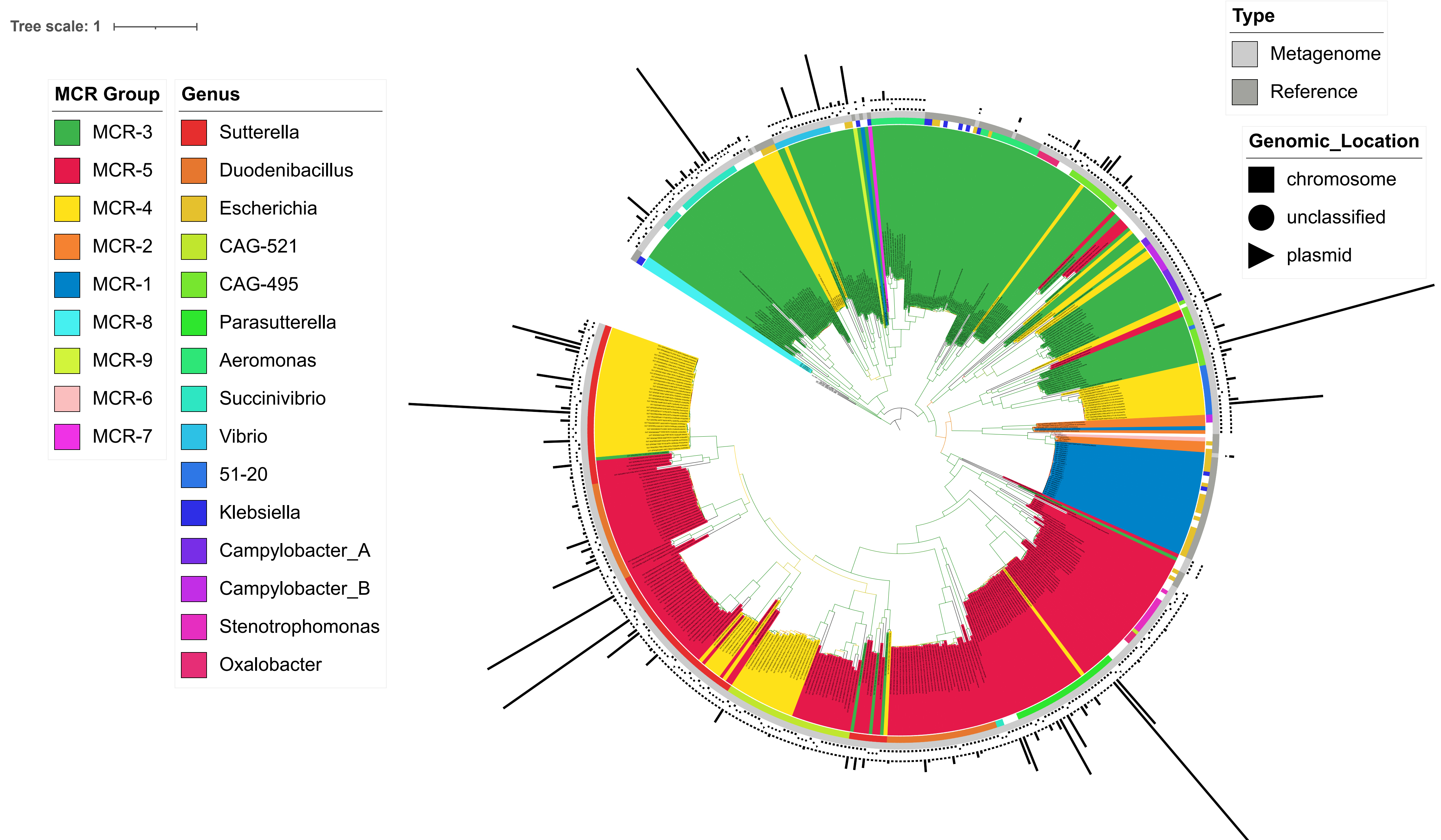
Tree scale: 1
MCR Group
MCR-3
MCR-5
MCR-4
MCR-2
MCR-1
MCR-8
MCR-9
MCR-6
MCR-7
Genus
Sutterella
Duodenibacillus
Escherichia
CAG-521
CAG-495
Parasutterella
Aeromonas
Succinivibrio
Vibrio
51-20
Klebsiella
Campylobacter_A
Campylobacter_B
Stenotrophomonas
Oxalobacter
Type
Metagenome
Reference
Genomic_Location
chromosome
unclassified
plasmid
